# Adipose-derived stem cell-released osteoprotegerin protects cardiomyocytes from reactive oxygen species-induced cell death

**DOI:** 10.1186/s13287-017-0647-6

**Published:** 2017-09-19

**Authors:** Jiyun Lee, Seahyung Lee, Chang Youn Lee, Hyang-Hee Seo, Sunhye Shin, Jung-Won Choi, Sang Woo Kim, Jong-Chul Park, Soyeon Lim, Ki-Chul Hwang

**Affiliations:** 1 Brain Korea 21 PLUS Project for Medical Science, Yonsei University, Seoul, Korea; 2 Institute for Bio-Medical Convergence, College of Medicine, Catholic Kwandong University, Gangneung, Gangwon-do Korea; 3 Department of Integrated Omics for Biomedical Sciences, Yonsei University, Seoul, South Korea; 4 Department of Environmental Engineering, Catholic Kwandong University, Gangneung-si, Gangwon-do Republic of Korea; 5 Cellbiocontrol Laboratory, Department of Medical Engineering, Yonsei University College of Medicine, Seoul, Republic of Korea

**Keywords:** Osteoprotegerin, Oxidative stress, Cardiomyocyte survival, Stem cell

## Abstract

**Background:**

The paracrine effect is likely the major mechanism of the adipose-derived stem cell (ASC)-mediated cardioprotective effect. However, the exact composition and nature of ASC-released paracrine factors remain elusive. In the present study, we examined the effect of osteoprotegerin (OPG), a stem cell-released decoy receptor for death ligand, on the survival of cardiomyocytes exposed to oxidative stress.

**Methods:**

The production of OPG from ASCs under oxidative stress was determined by ELISA and immunohistochemistry. The effects of OPG and the OPG-containing conditioned media of ASCs on the survival of cardiomyocytes were determined using a cell viability assay.

**Results:**

Hydrogen peroxide (H_2_O_2_) significantly increased OPG production from ASCs in vitro, and OPG production from the ASCs transplanted into the ischemia–reperfusion-injured heart was also observed. OPG significantly attenuated cardiomyocyte death in vitro. OPG-containing conditioned media of ASCs also significantly protected cardiomyocytes. Delivery of siRNA specific to OPG significantly decreased the OPG production of ASCs, and also offset the protective effect of the conditioned media of ASCs.

**Conclusions:**

Our study strongly suggests that OPG is one of the prosurvival factors released from ASCs that may contribute to the ASC-mediated cardioprotection and calls for further studies to elucidate detailed underlying mechanisms.

**Electronic supplementary material:**

The online version of this article (doi:10.1186/s13287-017-0647-6) contains supplementary material, which is available to authorized users.

## Background

Previous studies have reported the cardioprotective effect of adipose-derived stem cells (ASCs) [1, 2]. Evidence suggests that the regenerative effect of transplanted stem cells is mainly mediated by paracrine factors [3, 4], and stem cell-released factors varied depending on the source of stem cells and the stimuli they were exposed to [5, 6]. Therefore, it is important to identify soluble factors contributing to the beneficial effect (i.e., host cell protection) of stem cells under a specified condition because a clinically effective stem cell-based therapeutic strategy can be developed based on such information. Osteoprotegerin (OPG) is known to be produced by stem cells, fibroblasts, and endothelial cells [7]. OPG is a soluble decoy receptor that binds to tumor necrosis factor-related apoptosis-inducing ligand (TRAIL), thereby neutralizing TRAIL-mediated apoptotic signaling [8]. TRAIL initiates signaling cascade by binding to corresponding receptors such as TRAIL-R1, TRAIL-R2, TRAIL-R3, and TRAIL-R4 [9]. TRAIL-R1 and TRAIL-R2 can promote cell death signaling cascades, while TRAIL-R3 and TRAIL-R4 antagonize the TRAIL-R1 and TRAIL-R2-mediated apoptotic signaling by competitively binding to TRAIL [10]. In the present study, we examined the effect of OPG on the survival of cardiomyocytes exposed to oxidative stress.

## Methods

### Culture of ASCs and H9c2 cells

Four vials of ASCs from four different human donors (StemPro human adipose-derived stem cells) were purchased from Invitrogen (Carlsbad, CA, USA), and rat cardiomyoblast cell line H9c2 cells were purchased from ATCC (Manassas, VA, USA). ASCs and H9c2 cells were maintained according to the manufacturer’s instructions. We used low-glucose Dulbecco’s modified Eagle’s medium (DMEM; Gibco, Waltham, MA, USA) and high-glucose DMEM (Gibco) containing 10% fetal bovine serum (FBS; Gibco) and 1% penicillin–streptomycin (Gibco) for ASCs and H9c2 cells, respectively.

### Isolation rat neonatal ventricular cardiomyocytes

All experimental procedures for animal studies were approved by the committee for the care and use of laboratory animals of Catholic Kwandong University, and were performed in accordance with the committee’s guidelines and regulations (CKU01-2015-003-1). Neonatal rat ventricular cardiomyocytes were isolated and purified by enzymatic methods following a protocol published previously [11].

### Ischemia–reperfusion injury and transplantation of ASCs

Male 8-week-old Sprague–Dawley rats (250 g) were used. The animals were put under anesthesia with zoletil (30 mg/kg) and xylazine (10 mg/kg). The left anterior descending coronary artery was occluded using a 6-0 silk suture (Johnson & Johnson, New Brunswick, NJ, USA). After 60 min of occlusion, the left anterior descending coronary artery was released for reperfusion, followed by cell transplantation. For transplantation, 1 × 10^6^ cells were suspended in 30 μl PBS and injected to border zone using a 0.3-ml insulin syringe (BD Pharmingen, Franklin Lakes, NJ, USA).

### Statistical analyses

Quantitative data were expressed as the means ± SD of at least three independent experiments. For statistical analysis, one-way ANOVA with Bonferroni correction was performed using OriginPro 8 SR4 software (version 8.0951; OriginLab Corporation, Northampton, MA, USA) if there were more than three groups. *p* < 0.05 was considered statistically significant.

Additional methods are available in Additional file 1.

## Results

### Hydrogen peroxide induced apoptosis of cardiomyocytes

Hydrogen peroxide (H_2_O_2_), a member of endogenous reactive oxygen species (ROS), has been used to simulate oxidative stress [12, 13]. When H9c2 and primary cultured rat cardiomyocytes were treated with increasing concentration of H_2_O_2_ for 48 hours, the viability of both H9c2 (Additional file 2: Figure S1A) and primary (Additional file 2: Figure S1B) cardiomyocytes significantly decreased with H_2_O_2_ at a concentration of 100 μM or higher. However, primary cardiomyocytes were more resistant to ROS than H9c2 cardiomyocytes. Therefore, we mainly used H9c2 cardiomyocytes, which have been used as an alternative to primary cultured cardiomyocytes [14], to clearly demonstrate the prosurvival effect of OPG.

To confirm that the H_2_O_2_-induced cell death was oxidative stress mediated, the cells were pretreated with the well-established antioxidant *N*-acetyl-l-cystein (NAC) [15]. NAC pretreatment attenuated H_2_O_2_-induced activation of caspase 3 and 8 (Additional file 2: Figure S1C) and also significantly attenuated H_2_O_2_-induced decrease of H9c2 viability (Additional file 2: Figure S1D). Furthermore, NAC pretreatment decreased the number of apoptotic propidium iodide (PI)/Annexin V double-stained cells [16] (Additional file 2: Figure S1E). Interestingly, H_2_O_2_ concentration up to 200 μM did not decrease the viability of ASCs (Additional file 3: Figure S2).

### Oxidative stress increased OPG production from ASCs

**Fig. 1:**
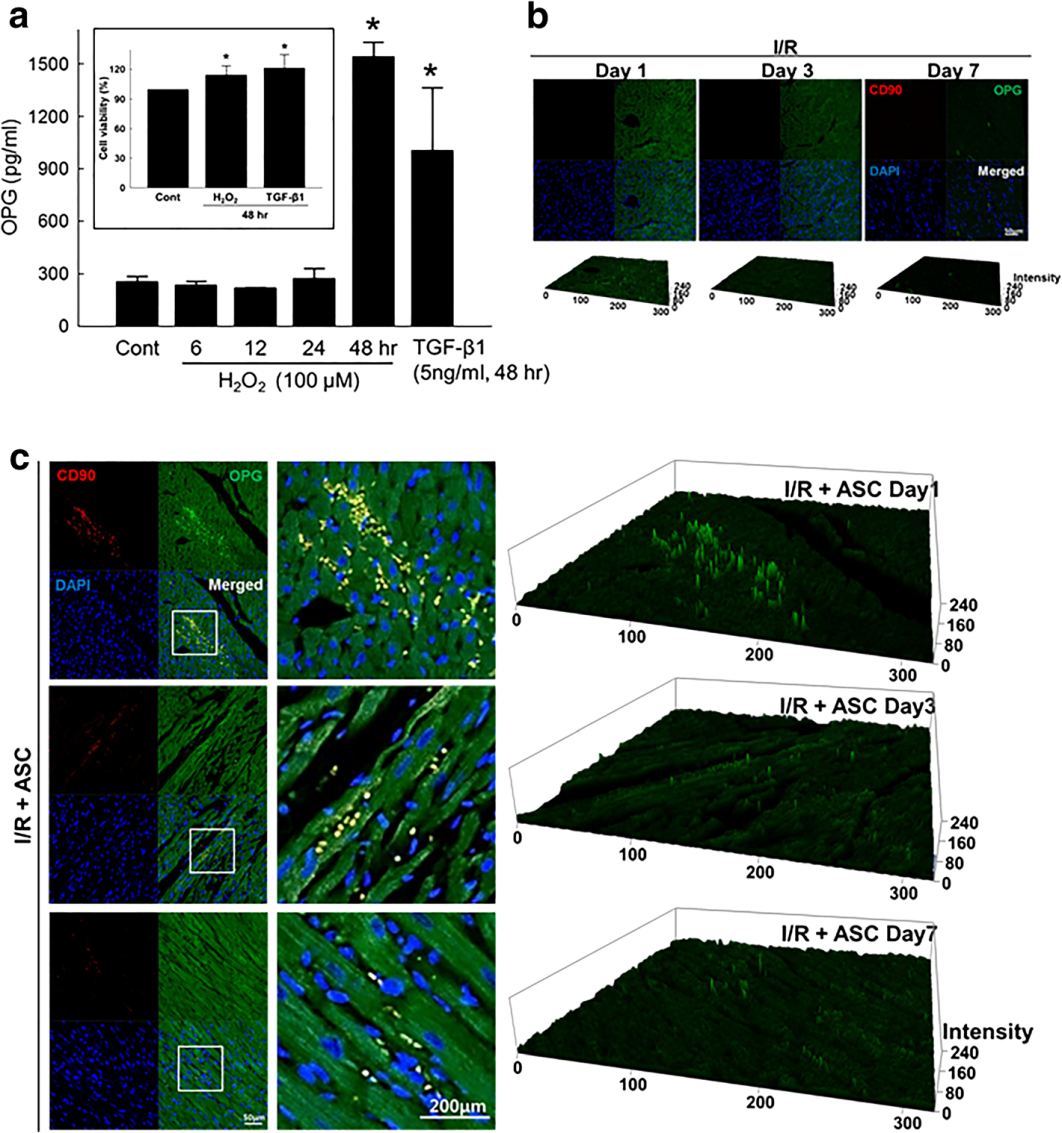
Oxidative stress induced OPG secretion from ACSs. (**a**) Amount of OPG secreted from ASCs exposed to H_2_O_2_ (100 μM) determined using an ELISA kit specific to human OPG. Conditioned media of ASCs exposed to H_2_O_2_ up to 48 hours were used for the analysis. TGF-β1 (5 ng/ml) was included as a soluble factor commonly present in damaged heart. Inset: viability of ASCs treated with H_2_O_2_ (100 μM) or TGF-β1 (5 ng/ml) for 48 hours. *p < 0.05 compared to untreated control. (**b**) Expression of OPG in ischemia–reperfusion (I/R)-injured rat heart examined by immunofluorescence staining. I/R-injured rat heart was collected 1, 3, and 7 days after the injury without ASC transplantation, and damaged heart was stained with antibodies specific to CD90 (red), a stem cell marker, and OPG (green). Nucleus was stained with DAPI (blue). (**c**) Expression of OPG in I/R-injured heart with ASC transplantation (1 × 10^6^ cells/head). Two-dimensional immunofluorescence images were converted to 2.5D topological view images for clear presentation of staining intensity among groups. ASC adipose-derived stem cell, OPG osteoprotegerin, TGF-β1 transforming growth factor beta 1 (Color figure online)

Both H_2_O_2_ and TGF-β1, another soluble factor commonly present in damaged heart [17], significantly increased the OPG production of ASCs without decreasing viability (Fig. 1a). Also, H_2_O_2_ significantly increased mRNA expression of OPG and TRAIL receptor 3 (TRAIL-R3) (Additional file 4: Figure S3), which attenuates TRAIL-induced apoptosis [18]. In the ischemia–reperfusion (I/R)-injured heart without ASC transplantation, OPG expression was marginal only at day 1 after I/R injury (Fig. 1b). However, in the ASC transplantation group, prominent OPG expression was observed in the vicinity of transplanted ASCs, which gradually decreased with time (Fig. 1c).

### OPG protected cardiomyocytes from ROS-induced cell death

**Fig. 2:**
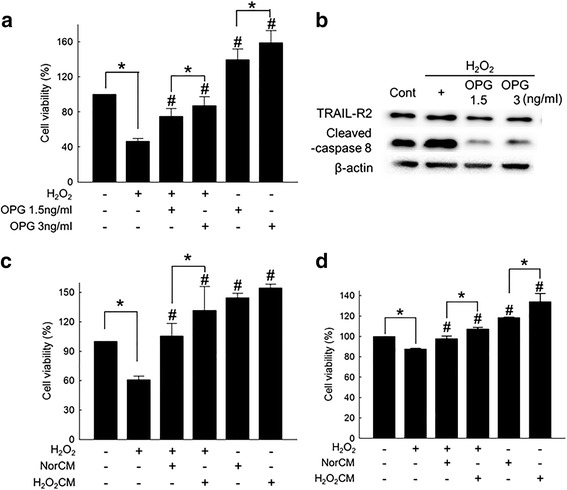
OPG protected cardiomyocytes from ROS-induced cell death. (**a**) Effect of OPG on H_2_O_2_-induced cell death of H9c2 examined using a cell counting kit. H9c2 cells were treated with 100 μM of H_2_O_2_ for 24 hours with or without OPG (1.5 and 3 ng/ml) treatment. *p < 0.05, #*p* < 0.05 compared to H_2_O_2_-treated group. (**b**) Expression of proapoptotic receptor TRAIL-R2 and activated (cleaved) caspase 8 in H9c2 cardiomyocytes exposed to H_2_O_2_ (100 μM) for 24 hours with or without OPG treatment (1.5 and 3 ng/ml) examined by western blot analysis. (**c**) Effect of ASC conditioned media with or without H_2_O_2_ conditioning on the viability of H9c2 cardiomyocytes exposed to H_2_O_2_. Normal conditioned media of ASC (NorCM) prepared by culturing ASCs for 48 hours and conditioned media with H_2_O_2_ conditioning (H_2_O_2_CM) prepared by culturing ASCs in the presence of 100 μM of H_2_O_2_ for 48 hours. H9c2 cells were cultured in NorCM or H_2_O_2_CM with or without addition of 100 μM of H_2_O_2_ for 24 hours. *p < 0.05, #*p* < 0.05 compared to H_2_O_2_-treated group. (**d**) Effect of ASC conditioned media with or without H_2_O_2_ conditioning on the viability of primary cardiomyocytes exposed to H_2_O_2_. The same experimental procedure used for H9c2 cardiomyocytes was applied to primary cardiomyocytes. *p < 0.05, #*p* < 0.05 compared to H_2_O_2_-treated group. OPG osteoprotegerin, TRAIL-R2 tumor necrosis factor-related apoptosis-inducing ligand receptor 2

To examine the effect of OPG on cardiomyocyte viability under oxidative stress, the cells were exposed to H_2_O_2_ with or without OPG (1.5 and 3 ng/ml) treatment. OPG treatment significantly protected from H_2_O_2_ (Fig. 2a) and suppressed activation of caspase 8 without affecting the expression of TRAIL-R2 (Fig. 2b). Both ASC conditioned media with or without 48 hours of H_2_O_2_ (100 μM) conditioning (designated as H_2_O_2_CM and NorCM, respectively) significantly attenuated H_2_O_2_-induced decrease of viability in both H9c2 (Fig. 2c) and primary (Fig. 2d) cardiomyocytes. Between NorCM and H_2_O_2_CM, H_2_O_2_CM was more effective to prevent H_2_O_2_-induced cardiomyocyte death.

### Downregulation of OPG offset cell protective effect of ASC conditioned media

**Fig. 3:**
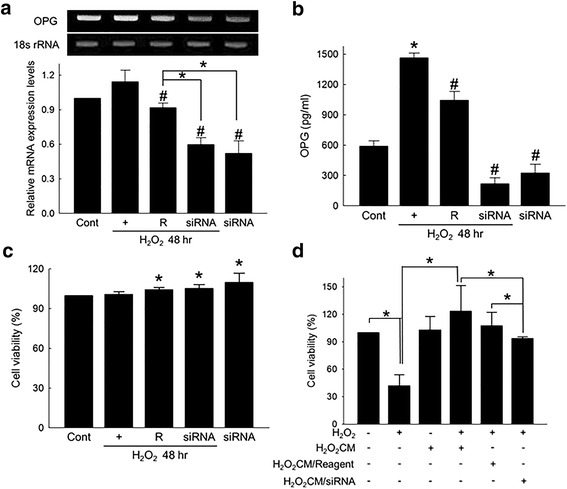
Downregulation of OPG offset protective effect of ASC conditioned media. (**a**) OPG-siRNA-mediated downregulation of OPG in H_2_O_2_-treated ASCs. ASCs were transfected with 20 nM of OPG-specific siRNA for 24 hours, and then exposed to 100 μM of H_2_O_2_ for an additional 48 hours. mRNA expression of OPG normalized by 18 s rRNA expression. *p < 0.05, #*p* < 0.05 compared to H_2_O_2_-treated group. (**b**) Effect of OPG-specific siRNA on the OPG production from ASCs exposed to H_2_O_2_. OPG-siRNA (20 nM)-transfected ASCs were cultured in the presence of 100 μM of H_2_O_2_ for 48 hours, and the amount of OPG in ASC culture media was determined using an OPG-specific ELISA kit. *p < 0.05 compared to untreated control, #*p* < 0.05 compared to H_2_O_2_-treated group. (**c**) Viability of OPG-siRNA-transfected ASCs exposed to 100 μM of H_2_O_2_. *p < 0.05 compared to H_2_O_2_-treated group. (**d**) Effect of three different types of H_2_O_2_CMs on H_2_O_2_-induced cell death of H9c2 cells. *p < 0.05. H_2_O_2_CM ASC conditioned media prepared by culturing ASCs with 100 μM of H_2_O_2_ for 48 hours, H_2_O_2_CM/Reagent conditioned media obtained using transfection reagent-treated ASCs, H_2_O_2_CM/siRNA conditioned media obtained using OPG-specific siRNA-transfected ASCs, OPG osteoprotegerin

To validate whether the cell protective effect of the ASC conditioned media was OPG mediated, siRNA specific to OPG was utilized. Delivery of OPG-specific siRNA to ASCs significantly attenuated OPG mRNA expression (Fig. 3a) and decreased OPG production (Fig. 3b). OPG-specific siRNA had no negative effect on the cell viability of ASCs (Fig. 3c). However, when siRNA specific to TRAIL-R3 was codelivered with OPG-specific siRNA, the viability of ASCs significantly decreased (Additional file 5: Figure S4). Finally, H_2_O_2_CM obtained from OPG-specific siRNA-treated ASCs showed decreased protective effect on cardiomyocytes exposed to H_2_O_2_ (Fig. 3d).

## Discussion

It has been reported that TRAIL was released from the postischemic heart soon after the onset of reperfusion [19]. Considering ROS production, a major cause of myocardial cell death in I/R injury [20], is an early event in myocardial reperfusion injury, TRAIL released at the onset of reperfusion may by associated with increased ROS and subsequent myocardial cell death in the I/R-injured heart. Therefore, we hypothesized that ASCs transplanted into damaged heart (i.e., I/R-injured heart where excessive oxidative stress causes cardiac cell death [21–23]) secrete OPG, and in turn the ASC-released OPG prevents host cardiomyocyte death by antagonizing TRAIL signaling, which has never been tested.

To test the hypothesis in vitro, we used 100 μM of H_2_O_2_ to simulate oxidative stress to which both host cardiomyocytes and transplanted ASCs are exposed in vivo. In our study, H_2_O_2_ induced cardiomyocyte death via oxidative stress-mediated activation of apoptosis, confirming the validity of our experimental setting (Additional file 2: Figure S1). When ASCs were exposed to H_2_O_2_ or transplanted to I/R-injured heart, the production of OPG was significantly increased (Fig. 1), suggesting that ASCs exposed to a hostile microenvironment, such as I/R-injured heart, can produce OPG for the first few days following transplantation. Further experiments showed that OPG can suppress ROS-induced apoptosis of cardiomyocytes (Fig. 2a, b) and demonstrated that H_2_O_2_ conditioning of ASCs enhanced the protective effect of conditioned media possibly by increasing OPG production from ASCs (Fig. 2c, d). Experiments using OPG-specific siRNA indicated that the cell protective effect of ASC conditioned media was indeed OPG mediated (Fig. 3).

One interesting observation was that ASCs were less sensitive to H_2_O_2_ compared to cardiomyocytes (Additional file 3: Figure S2). This might have something to do with the increased OPG and TRAIL-R3 expression under oxidative stress (Additional file 4: Figure S3) because cotransfection of siRNAs specific to OPG and TRAIL-R3 significantly decreased the viability of ASCs exposed to H_2_O_2_ (Additional file 5: Figure S4). This also suggested that OPG and TRAIL-R3 may compensate each other in terms of suppressing TRAIL-mediated cell death in ASCs. Nevertheless, without any further experimental evidence, this can only be speculated at this point. We are currently working to elucidate more detailed underlying mechanisms of OPG-mediated cardiomyocyte protection and to validate and confirm the findings of this study in vivo for further studies.

## Conclusion

In the present study we provided evidence that OPG is an ASC-released soluble factor that can protect cardiomyocytes from ROS-induced apoptosis for the first time. The results of this study warrant further studies to fully elucidate the exact nature of stem cell-released paracrine factors and may help us to design an effective stem cell-based therapeutic strategy in the future.

## Additional files

Additional file 1: Describes additional methods including primers used for RT-PCR. (DOCX 34 kb)

Additional file 2: **Figure S1.** Showing ROS-induced cardiomyocyte death. (**A**) Viability of H9c2 cardiomyocytes exposed to increasing concentration of H_2_O_2_ for 24 hours. *p < 0.05 compared to untreated control. (**B**) Effect of H_2_O_2_ on viability of primary cardiomyocytes. *p < 0.05 compared to untreated control. (**C**) Expression of activated (cleaved) caspase 3 and 8 in H9c2 cardiomyocytes exposed to H_2_O_2_ (100 μM) for 24 hours with or without antioxidant *N*-acetyl-l-cystein (NAC) (1.5 mM). (**D**) Viability of H9c2 cardiomyocytes exposed to H_2_O_2_ (100 μM) for 24 hours with or without antioxidant NAC (1.5 mM). *p < 0.05. (**E**) Flow cytometry analysis of H9c2 cardiomyocytes exposed to H_2_O_2_ (100 μM) for 24 hours with or without antioxidant NAC (1.5 mM). PI propidium iodide. (PDF 237 kb)

Additional file 3: **Figure S2.** Showing effect of H_2_O_2_ on ASC viability. *p < 0.05 compared to control. (PDF 36 kb)

Additional file 4: **Figure S3.** Showing ROS-induced OPG and TRAIL-R3 expression in ASCs. *p < 0.05 compared to corresponding control of each group. (PDF 160 kb)

Additional file 5: **Figure S4.** Showing that OPG and TRAIL-R3 contribute to the survival of ASCs exposed to ROS. (**A**) Expression of TRAIL-R3 mRNA 24 hours after transfection of siRNA specific to TRAIL-R3. 18 s-rRNA used as internal control. (**B**) Effect of OPG and/or TRAIL-R3 downregulation on the viability of ASCs exposed ROS. Cells transfected with OPG siRNA and/or TRAIL-R3 siRNA for 24 hours and then exposed to H_2_O_2_ (100 and 200 μM) for an additional 24 hours. *p < 0.05 compared to untreated control. (PDF 87 kb)

## Abbreviations

ASC: Adipose-derived stem cell
DMEM: Dulbecco’s modified Eagle’s medium
ELISA: Enzyme-linked immunosorbent assay
FBS: Fetal bovine serum
H_2_O_2_CM: ASC conditioned media with H_2_O_2_ conditioning
I/R: Ischemia–reperfusion
NAC: *N*-acetylcystein
NorCM: ASC conditioned media without H_2_O_2_ conditioning
OPG: Osteoprotegerin
PBS: Phosphate-buffered saline
ROS: Reactive oxygen species
TGF-β1: Transforming growth factor beta 1
TNF: Tumor necrosis factor
TRAIL: Tumor necrosis factor-related apoptosis-inducing ligand
TRAIL-R1–R4: TRAIL receptor 1–4
