# Supplementary material for: Adipose-derived stem cell-released osteoprotegerin protects cardiomyocytes from reactive oxygen species-induced cell death
Source: Stem Cell Res Ther. 2017 Sep 19;8:195. doi: 10.1186/s13287-017-0647-6 (PMC5606035; doi:10.1186/s13287-017-0647-6)
Supplement: Supplementary file 1 — Describes additional methods including primers used for RT-PCR. (DOCX 34 kb) [file 13287_2017_647_MOESM1_ESM.docx]

**Additional File 1: additional methods**

**Cell viability assay**

After treating cells, EZ-cytox reagent (DoGen, Seoul, Korea) was added to culture media and incubated at 37°C for 2 hrs. The absorbance at 450 nm was determined using a microplate reader.

**Annexin V/PI assay**

Apoptosis was measured using a FITC Annexin V Apoptosis Detection Kit I (BD Pharmingen, Franklin Lakes, USA) according to the manufacture’s instruction.

**siRNA transfection**

OPG and TRAIL-R3 siRNA were purchased from Bioneer (Daejeon, Korea) and Integrated DNA Technologies (Coralville, USA), respectively. Transfection of siRNAs was performed using TransIT-X2 reagent (Mirus Bio, Madison, USA).

**ELISA**

Human OPG ELISA kits were purchased from AbFrontier (Seoul, Korea) and ELISA was performed according to the manufacturer’s instruction.

**Immunohistochemistry**

Heart tissues were fixed overnight with 10% (v/v) neutral-buffered formaldehyde and embedded in paraffin. The sections were stained for DAPI (Sigma-Aldrich, St. Louis, USA), CD90 (Santa Cruz Biotechnology, Dallas, USA), and OPG (Abcam, Cambridge, UK) by confocal laser scanning microscope LSM700 (Carl Zeiss, Oberkochen, German).

**Reverse transcriptase polymerase chain reaction (RT-PCR)**

Five hundred ng of total RNA was used to synthesize cDNA. The primer sequences are the followings.

**
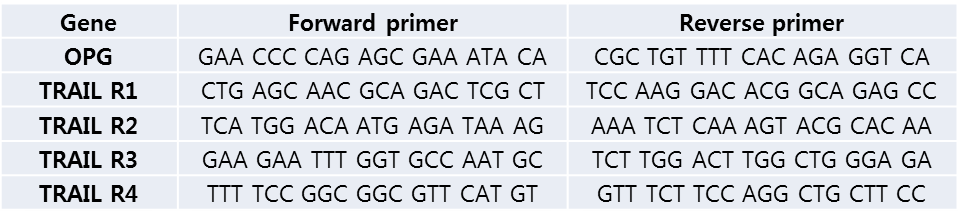
**

**Western blot analysis**

The following antibodies were used for western blot; Caspase 3 (Millipore, Billerica, USA), caspase 8 (Abcam), TRAIL-R2 (Santa Cruz Biotechnology), and β-actin (Sigma-Aldrich).
