# Supplementary figures and images for: Adipose-derived stem cell-released osteoprotegerin protects cardiomyocytes from reactive oxygen species-induced cell death
Source: Stem Cell Res Ther. 2017 Sep 19;8:195. doi: 10.1186/s13287-017-0647-6 (PMC5606035; doi:10.1186/s13287-017-0647-6)

**Figure S1.**

**A**

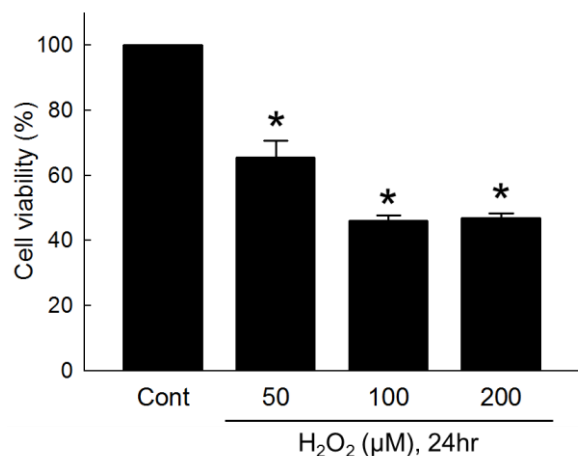

**B**

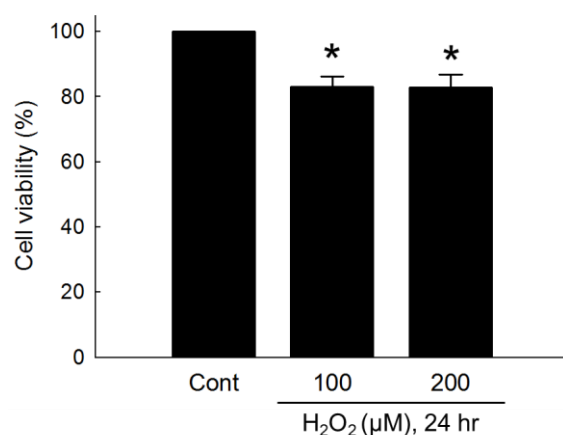

**C**

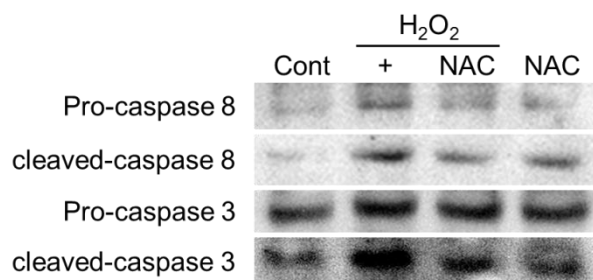

**D**

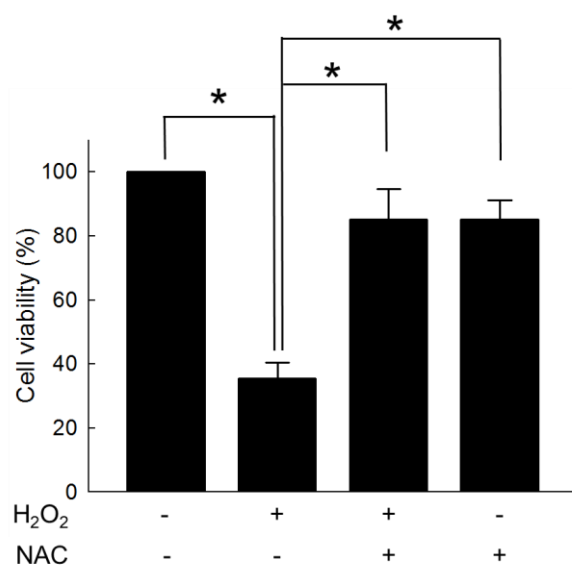

**E**

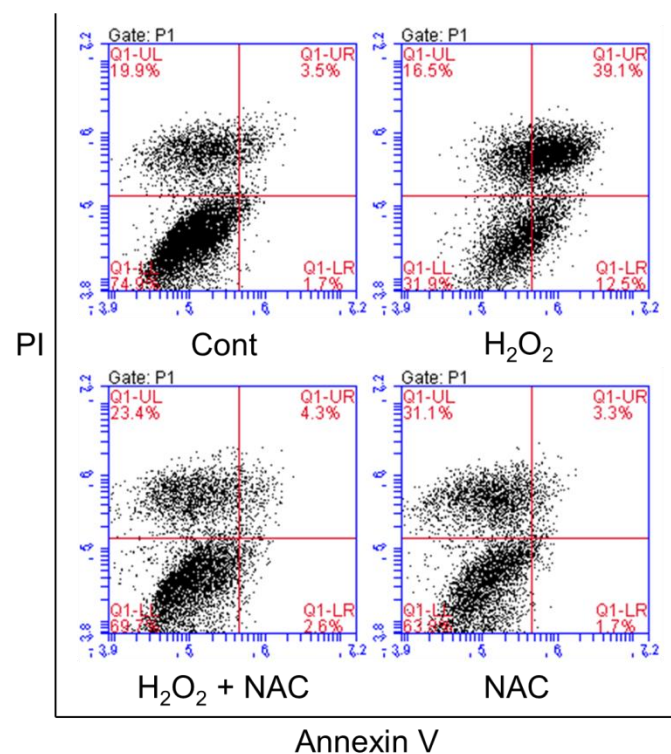

Supplement: Supplementary file 2 — Figure S1. Showing ROS-induced cardiomyocyte death. (A) Viability of H9c2 cardiomyocytes exposed to increasing concentration of H2O2 for 24 hours. *p < 0.05 compared to untreated control. (B) Effect of H2O2 on viability of primary cardiomyocytes. *p < 0.05 compared to untreated control. (C) Expression of activated (cleaved) caspase 3 and 8 in H9c2 cardiomyocytes exposed to H2O2 (100 μM) for 24 hours with or without antioxidant N-acetyl-l-cystein (NAC) (1.5 mM). (D) Viability of H9c2 cardiomyocytes exposed to H2O2 (100 μM) for 24 hours with or without antioxidant NAC (1.5 mM). *p < 0.05. (E) Flow cytometry analysis of H9c2 cardiomyocytes exposed to H2O2 (100 μM) for 24 hours with or without antioxidant NAC (1.5 mM). PI propidium iodide. (PDF 237 kb) [file 13287_2017_647_MOESM2_ESM.pdf]

**Figure S2.**

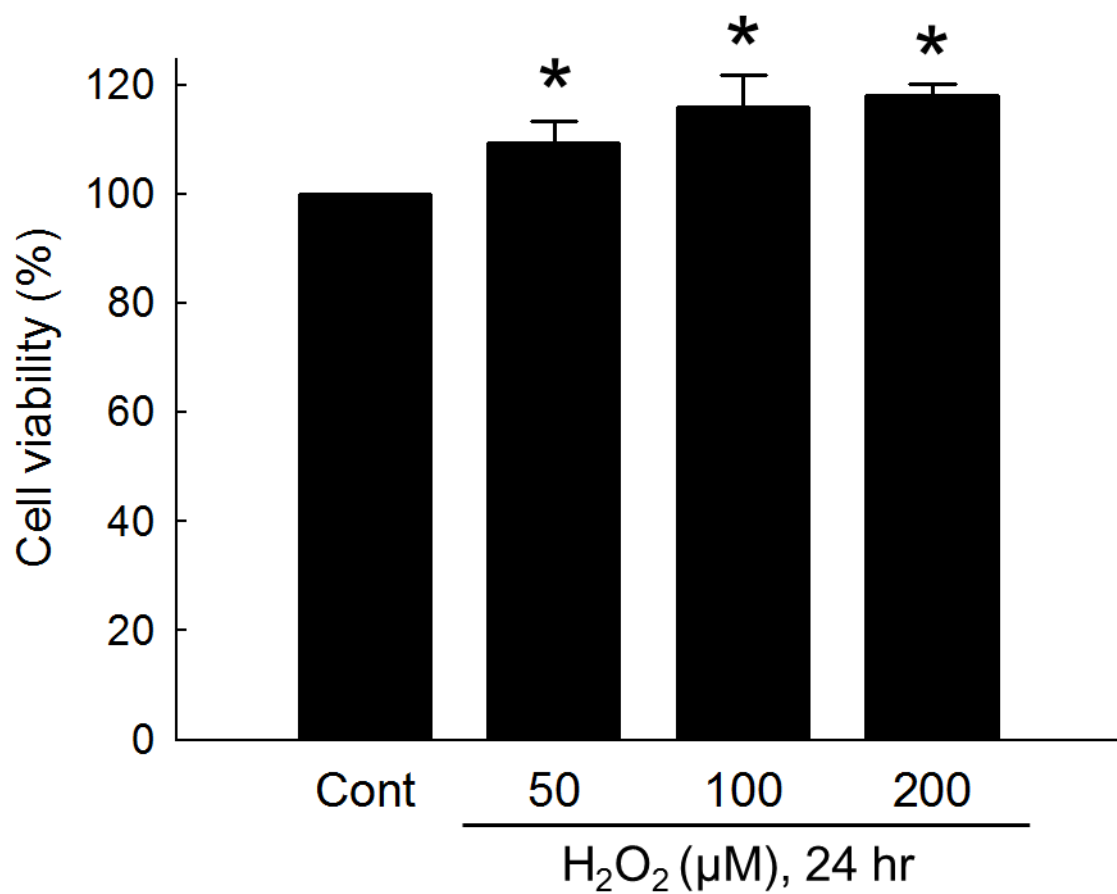

Supplement: Supplementary file 3 — Figure S2. Showing effect of H2O2 on ASC viability. *p < 0.05 compared to control. (PDF 36 kb) [file 13287_2017_647_MOESM3_ESM.pdf]

**Figure S3.**

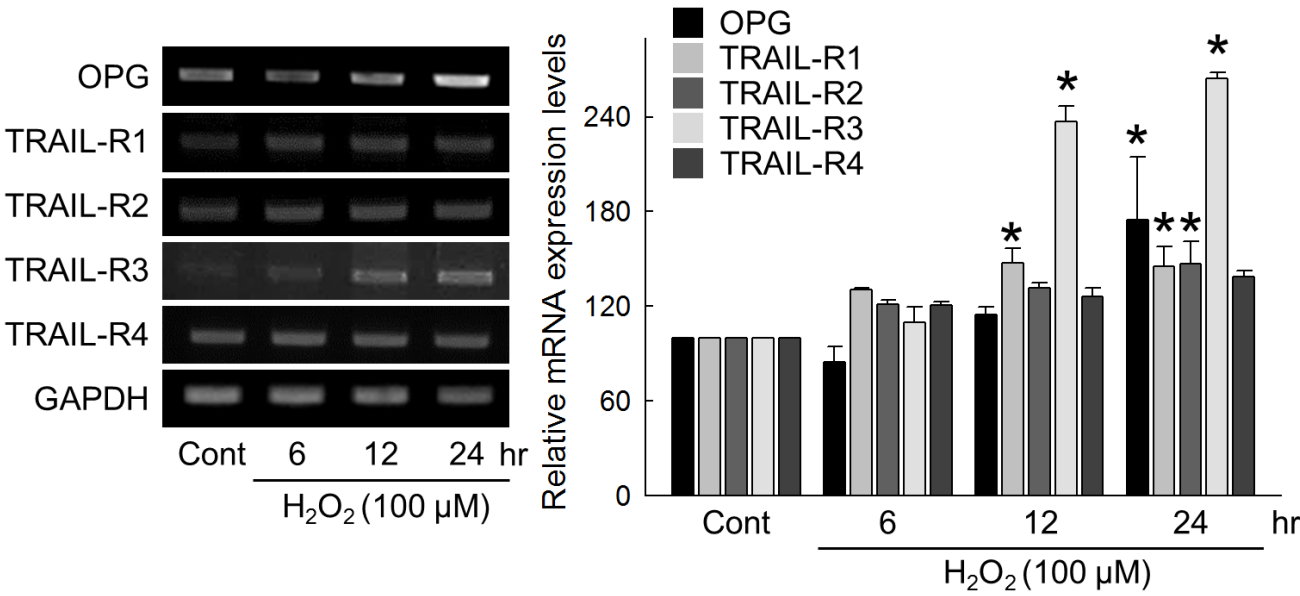

Supplement: Supplementary file 4 — Figure S3. Showing ROS-induced OPG and TRAIL-R3 expression in ASCs. *p < 0.05 compared to corresponding control of each group. (PDF 160 kb) [file 13287_2017_647_MOESM4_ESM.pdf]

Figure S4.

A

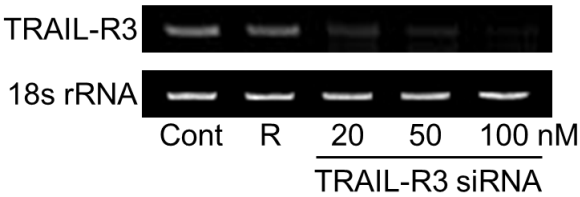

B

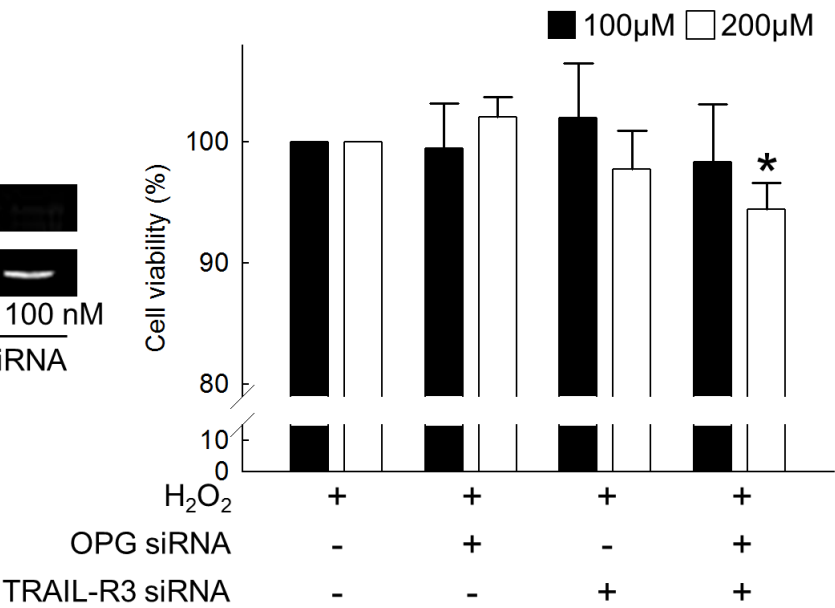

Supplement: Supplementary file 5 — Figure S4. Showing that OPG and TRAIL-R3 contribute to the survival of ASCs exposed to ROS. (A) Expression of TRAIL-R3 mRNA 24 hours after transfection of siRNA specific to TRAIL-R3. 18 s-rRNA used as internal control. (B) Effect of OPG and/or TRAIL-R3 downregulation on the viability of ASCs exposed ROS. Cells transfected with OPG siRNA and/or TRAIL-R3 siRNA for 24 hours and then exposed to H2O2 (100 and 200 μM) for an additional 24 hours. *p < 0.05 compared to untreated control. (PDF 87 kb) [file 13287_2017_647_MOESM5_ESM.pdf]
